# Epstein-Barr Virus: The Path from Association to Causality for a Ubiquitous Human Pathogen

**DOI:** 10.1371/journal.pbio.1001939

**Published:** 2014-09-02

**Authors:** Bill Sugden

**Affiliations:** McArdle Laboratory for Cancer Research, University of Wisconsin, Madison, Madison, Wisconsin, United States of America

## Abstract

Epstein-Barr virus is notorious for causing multiple kinds of cancer. It has also been increasingly linked to multiple sclerosis. What evidence now supports or can be sought potentially to strengthen this linkage?

Epstein-Barr virus (EBV), a herpes virus, is now accepted as a bona fide human tumor virus and has been found to be a risk factor for the development of multiple sclerosis (MS). Epidemiological studies and molecular virology have been combined to establish EBV's causal roles in several lymphomas and carcinomas. The success of these combined approaches illustrates what insights will be needed to confirm or refute EBV as a cause of MS.

## Epstein-Barr Virus Causes Burkitt Lymphoma

**Figure 1:**
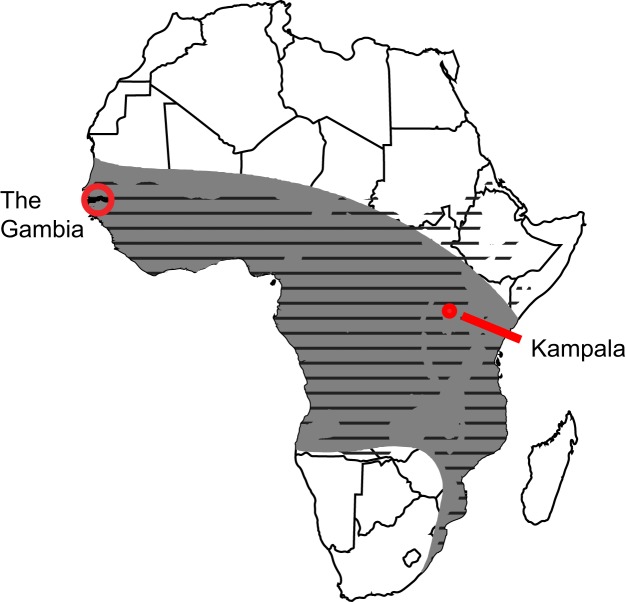
The Burkitt lymphoma belt overlaps the regions of Africa in which malaria is holoendemic. The grey belt shown on the map of Africa represents the areas in which Denis Burkitt's surveys found Burkitt lymphoma to be endemic. The black horizontal stripes represent regions of Africa in which the temperatures and rainfall support holoendemic malaria. Kampala is where Denis Burkitt treated his patients; the Gambia is one site where the role of malaria as a cofactor for causing Burkitt lymphoma was demonstrated.

EBV causes Burkitt lymphoma, and the tale of how this association was first discovered is marvelous and warrants retelling [1]. In the 1950s, Denis Burkitt, a perceptive surgeon working in Kampala, Uganda, recognized a childhood tumor as a new clinical entity. It is now known as Burkitt lymphoma. In order to understand the distribution of this tumor, Burkitt conducted several epidemiological surveys extraordinary both for their simplicity and insights. With a grant of 25 pounds, he mailed to dispensaries throughout Central Africa leaflets depicting children with the lymphoma and a questionnaire asking if patients with similar symptoms had been treated. With a larger, later grant of 240 pounds, he purchased a used vehicle and with two colleagues traveled 10,000 miles throughout southeastern Africa, interviewing physicians in 60 hospitals. Finally, he traveled to West Central Africa to interview doctors there to gauge their experience with such childhood tumors. His surveys identified the geographical extent of Burkitt lymphoma as a lymphoma belt stretching across Central Africa (Figure 1). He and his colleagues searched for defining features of this geography and recognized that its rainfall and temperature range characterized the conditions most favorable for holoendemic malaria (Figure 1). This insight led them to hypothesize that the lymphoma was caused by an infectious agent carried by an insect vector, as the *Anopheles* mosquito carries the malarial parasite. While the hypothesis was wrong in detail, it provided the impetus to Anthony Epstein and his two colleagues, Bert Achong and Yvonne Barr, to collaborate with Denis Burkitt to search for and identify EBV in 1964 [2]. The hypothesis also focused attention on malaria as a potential cofactor in causing Burkitt lymphoma.

In the 1970s, the World Health Organization conducted a prospective survey in East Central Africa by collecting sera from 42,000 children eight years of age or younger; by 1982,16 of these had developed Burkitt lymphoma [3],[4]. When compared to matched, control children, those children who did develop the tumor had higher titers of antibodies to certain EBV-encoded proteins months before they showed symptoms of the tumor. This finding indicated that abnormally high titers of antibodies to some EBV antigens constituted a 30-fold risk factor for the development of Burkitt lymphoma.

One additional insight was provided by following children in the Gambia on the West Coast of Africa, where malaria is holoendemic and Burkitt lymphoma common. Children during the acute phase of malaria were found to have more cells infected with EBV in their blood than when convalescent, making it likely both that malaria inhibited immune responses to EBV and yielded more cells at risk to evolve into Burkitt lymphoma [5].

These multiple associations of EBV, Burkitt lymphoma, and malaria have been buttressed by molecular studies of the virus. Anthony Epstein's identification of EBV allowed detection of its DNA in cells derived from Burkitt lymphomas by Harald zur Hausen [6]. Related studies of all tumors associated with EBV have demonstrated over the years that the viral DNA is present in most or all of the tumor cells. The virus thus is situated where it can either benefit from or benefit the tumor. EBV was shown also to infect resting B cells and to induce and maintain their proliferation in cell culture [7]. This phenotype is striking: no other virus has been identified that can infect nondividing B cells and drive their proliferation as efficiently as can EBV. It clearly calls to mind a potential to be tumorigenic. However, about 90% of all people are infected lifelong with EBV, and only 200,000 or so of us develop EBV-associated tumors each year. A chromosomal abnormality of Burkitt lymphoma cells has helped to explain the rarity of this particular tumor among infected people. The tumor cells in patients where the tumor is endemic almost always have a chromosomal translocation juxtaposing the c-myc proto-oncogene with one of the three immunoglobulin loci, allowing the translocated c-myc allele to be expressed constitutively [8],[9]. This translocation is rare, peculiar to B cells in which immunoglobulins are expressed, and surely contributes to the evolution of Burkitt lymphomas.

**Figure 2:**
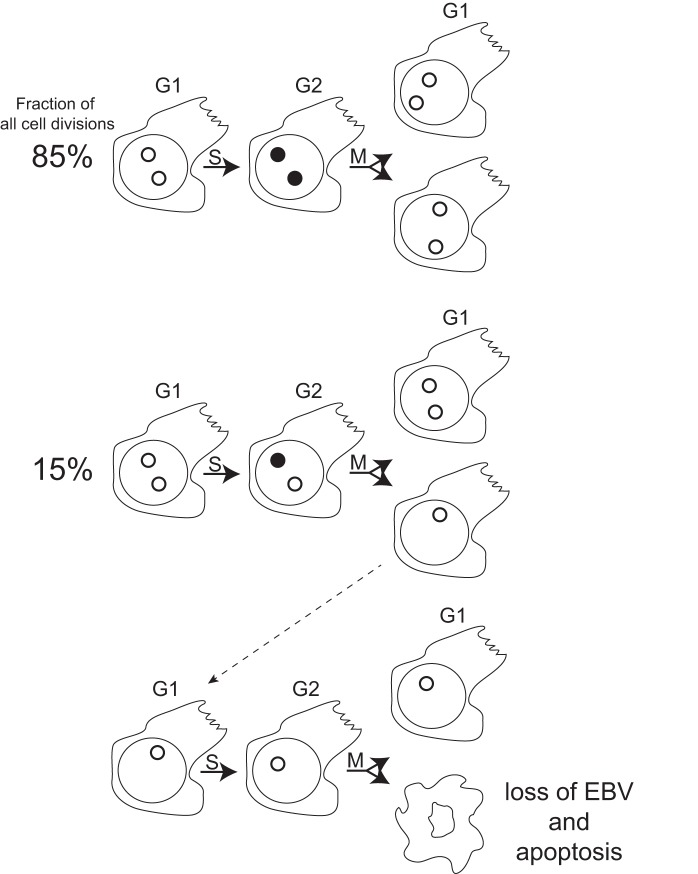
Defects in the synthesis of EBV DNA lead to its loss in proliferating cells. Shown diagrammatically are lymphoid cells with two molecules of EBV DNA (open circles) present in the cells on the left in the G1 phase of the cell cycle. During S phase, ∼85% of the viral DNAs are synthesized and remain localized together (black circles); 15% of the viral DNAs fail to be synthesized (open circle). The newly synthesized viral DNAs are segregated during M phase to the daughter cells faithfully 88% of the time as shown in the pairs of daughters on the right. The defects in viral DNA synthesis lead to some daughter cells having fewer viral DNA molecules than did their parents and inevitably to some daughter cells having none as shown for the progeny at the bottom of the figure. Cells dependent on EBV for survival functions as found for EBV-positive lymphomas will die by apoptosis on losing the viral genome.

How were molecular studies used to discriminate between the virus contributing to and maintaining the tumor or residing passively in its cells? This conundrum was addressed serendipitously through studies of EBV's genome. The viral DNA is maintained in cells as an extrachromosomal replicon with a wide distribution in the number of viral plasmids per cells. Live-cell imaging of these plasmids showed that about 15% of them failed to be synthesized in each cell cycle in all cell types examined [10]. This failure means that proliferating cells inevitably lose EBV DNA (Figure 2). A population of cells, for example, those in a tumor in vivo, will retain EBV DNA only if the virus provides the tumor cells one or more selective advantages allowing them to outgrow their sisters that lose EBV. This interpretation has been supported by examining the fate of engineered lymphoma cells from which the viral plasmids could be forcibly evicted; they died by apoptosis as they lost EBV [11].

A therapeutic finding has also documented EBV as maintaining lymphomas and underscored the role of immune defects in fostering EBV's tumorigenesis. One kind of malignancy associated with EBV arises in transplant recipients who are immunosuppressed to allow them to accept their grafts. On the order of 1% of these recipients develop post-transplant lymphoproliferative disorder (PTLD). The proliferating cells usually are EBV-infected B cells that can evolve into fatal lymphomas. The patients can sometimes be treated merely by reducing their immunosuppression so that their own immune responses control and eliminate the EBV-infected cells. However, they also can be successfully treated with infusions of T cells from the donor educated in vitro to kill their syngeneic, EBV-infected B cells [12]. These findings demonstrate that EBV's induced proliferation of infected B cells needs to be controlled by the host's immune responses; in their absence, the infected cells can evolve to become tumors.

## Epstein-Barr Virus Causes Nasopharyngeal Carcinoma

More than 50 years of epidemiology and molecular virology focused on EBV have yielded compelling data to show that EBV causes EBV-positive lymphomas. Much epidemiology also associates EBV with two kinds of carcinomas, but the biology of these tumors makes their molecular study difficult. The acceptance of EBV as causing, for example, nasopharyngeal carcinoma (NPC), has thus come in part by its also being accepted as causing several lymphomas. The findings associating EBV with NPC include the detection of high titers of antibodies of the immunoglobulin A (IgA) class to certain EBV antigens both in tumor patients and before people develop the tumor [13],[14]. In the latter case, Yi Zeng and his colleagues organized a large prospective survey in southern China and found that high titers of IgA anti-EBV antibodies correlated with a 30-fold increased risk of people developing NPC. Other epidemiological studies have shown that alleles of the major histocompatibility locus correlate with an increased risk of developing NPC, too [15],[16].

A clever experiment by George Klein allowed a clear molecular analysis of NPC tumors. Fresh biopsies were passed in immunodeficient nude mice, allowing the tumor cells to proliferate and the supporting human stroma to be replaced with murine cells. This and other experiments have documented that effectively all the epithelial tumor cells contain EBV DNA as plasmids and express viral products [17],[18]. When these cells are placed in culture, they usually lose the viral DNA, indicating that the selective advantages EBV must confer on NPC cells to be retained in vivo do not apply in vitro. In addition, no transformation assays for primary epithelial cells have been developed for EBV, so the phenotypes it might provide newly infected epithelial cells remain unknown. Finally, treatment of NPC patients with their own cytotoxic T cells expanded in vitro to recognize EBV-encoded products can be therapeutically beneficial, particularly when the tumors are localized [19]. This finding at the very least shows that EBV marks the NPC cells as targets for their being killed.

## Epstein-Barr Virus Causes Additional Types of Cancers

Most viral pathogens cause a specific disease that reflects the specific type of cell that virus infects. During the last few decades, EBV has been found to infect an increasing variety of cell types and correspondingly become associated causally with an increasing number of cancers. The evidence for this causality varies but always includes the presence of viral genomes in the tumor cells. EBV is now accepted to cause between 40% and 50% of Hodgkin disease, a B cell lymphoma marked by its absence of immunoglobulin expression [20]. B cells that fail to rearrange their immunoglobulin genes productively to allow one to be expressed die by apoptosis. EBV permits such B cells to survive, thus explaining one contribution EBV makes to their evolution into lymphomas [21]. EBV can infect T cells and natural killer (NK) cells. A spectrum of pathologies are found in patients with T/NK cell lymphoproliferative diseases, many of which progress to EBV-positive lymphomas [22]. EBV is now thought also to cause between 5%–10% of gastric carcinomas [23], a tumor commonly associated causally with infection by the bacterium, *Helicobacter pylori* [24]. There are close to 1 million new cases of this cancer in the world each year, so EBV's role in causing 5%–10% of these tumors is significant. These tumors tend to lose EBV upon being placed into cell culture, but Kenzo Takada and his colleagues have shown that the reintroduction of EBV into these cells fosters their growth, indicating one advantage EBV likely provides gastric carcinomas in vivo [25].

Our appreciation that EBV plasmid genomes are retained in proliferating cells only if the virus provides those cells a selective advantage supports EBV's contributing causally to all of these associated cancers. In addition for many of them, both epidemiology and molecular studies have shown that atypical immune responses to EBV-encoded antigens precede clinical recognition of EBV-associated tumors, and immunotherapies directed against EBV-encoded proteins can be effective in treating these tumors. These findings, when coupled with the extensive studies of tumor cells in vitro [26], have led the medical community to accept EBV's causal roles in these tumors.

## Epstein-Barr Virus May Contribute Causally to Multiple Sclerosis

What data associates EBV with MS, a disease that afflicts 1 to 2 million people today? To address this question, it is important to distinguish EBV's cancers from MS. These tumors represent proliferating, infected cells; MS is a neurodegenerative disease resulting from demyelination leading to neuronal conduction blocks and potentially neuronal cell death. While EBV is found in its associated tumor cells, there is no reason to think it is in the myelin-producing glial cells or in the neurons whose axons are wrapped with myelin. Rather, it appears that the host's immune response to EBV underlies the possible association of infection with EBV and a host's risk of developing MS.

It has been appreciated for years that people who develop infectious mononucleosis, a benign, self-limiting B cell proliferation, have an increased risk of developing MS [27]. EBV causes infectious mononucleosis, which usually occurs when adolescents are infected with the virus for the first time. Most people in the world will be first infected at a younger age and will not develop infectious mononucleosis and its associated risk for MS. More recently, higher titers of antibodies to EBV-encoded nuclear proteins have been found to correlate with the risk of developing MS [28]. In addition, another prospective study looking for people who developed MS without first being infected with EBV could find no such patients. Rather, people who were initially uninfected in all cases became infected prior to developing MS [29]. No other virus has been found so far to share these immune-related correlations with MS as does EBV.

One possible explanation for EBV's being causally associated with MS comes from a well-established genetic contribution to MS. People with certain human leukocyte antigen (HLA) alleles such as DRB1*1501 have significantly increased risk of developing MS [30]. Patients with MS have increased levels of CD4+ T cells that recognize one viral protein, Epstein-Barr nuclear antigen 1 (EBNA1), and can kill EBV-positive cells. A fraction of these CD4+ T cells also recognize myelin-derived peptides [31]. If any of the HLA alleles that are associated with increased risk of acquiring MS were found to mediate the cross recognition of EBNA1 and myelin, then this finding would lend support to EBV contributing directly to MS. CD4+ T cells primed to kill EBV-infected cells might recognize myelin-producing cells. Of course, such alleles may not exist.

## From Association to Causality for Epstein-Barr Virus and Multiple Sclerosis: What Is Needed?

In contrast to EBV and its associated cancers, any evidence supporting a causal role for EBV in MS is unlikely to come from an analysis of the glial or neuronal cells affected by the disease. Elucidating a possible relationship of the immune response to EBV infection and the development of MS should illuminate EBV's potential contribution to MS. The most compelling evidence for such a contribution, though, would come by eliminating EBV as a human pathogen (Box 1). Would eradication of EBV lead to a concomitant reduction in the incidence of MS? We can hope so and we know now that eradicating EBV would lead to the eradication of multiple kinds of cancers worldwide.

**Box 1. Eliminating Epstein-Barr Virus–Associated Diseases**

Those of us who began studying EBV in the 1960s and '70s worked in an exciting time in which there was the prospect of eliminating small pox and polio, two major, worldwide diseases caused by viruses, by vaccination. More recently, we have seen the development and use of subunit vaccines against hepatitis B virus and human papilloma viruses to block their infection to prevent multiple, prevalent cancers. These vaccines represent profound advances for public health across the world. However, only one vaccine has been developed and used successfully to prevent or treat infections with a human herpesvirus; that is for varicella zoster virus, the cause of chicken pox and shingles [32],[33]. This is a live vaccine, and live vaccines for a human tumor virus such as EBV are impractical. We need now either to develop subunit vaccines [34] or pharmacological inhibitors that are effective for EBV. We know that inhibitors that force the loss of EBV from cells should be therapeutically beneficial for EBV's associated tumors [11]. A vaccine to block its infection would have the advantage of being the ultimate test to determine if EBV contributes causally to MS.
